# Emerging therapeutic strategies to enhance HDL function

**DOI:** 10.1186/1476-511X-10-175

**Published:** 2011-10-10

**Authors:** Santiago Redondo, José Martínez-González, Concha Urraca, Teresa Tejerina

**Affiliations:** 1 Department of Pharmacology, School of Medicine, Universidad Complutense de Madrid, Spain; 2 Service of Hematology, Hospital Nuestra Señora de Sonsoles, Ávila, Spain; 3 Cardiovascular Research Center (CSIC-ICCC), IIB-Sant Pau, Barcelona, Spain

## Abstract

Epidemiologic studies indicate a strong inverse correlation between plasma levels of high-density lipoproteins (HDL) and cardiovascular disease (CVD). The most relevant cardioprotective mechanism mediated by HDL is thought to be reverse cholesterol transport (RCT). New insights in HDL biology and RCT have allowed the development of promising agents aimed to increase HDL function and promote atherosclerosis regression. In this regard, apo-AI analogs and CETP inhibitors dalcetrapib and anacetrapib have aroused a great interest and opened new expectations in the treatment of CVD.

## Introduction

Low levels of high-density lipoproteins (HDL) are among the strongest, statistically independent risk factors for cardiovascular disease (CVD). It has been estimated that a 1 mg/dL increase of HDL-cholesterol in plasma results in a 2-3% decrease in CVD risk [1]. The most widely accepted mechanism for this HDL protective effect is the reverse cholesterol transport (RCT). RCT refers to the mechanism by which cholesterol excess is transported from cells of extrahepatic tissues and carried back to the liver, where it can be eliminated or recycled. There has been a rising interest in the physiology and pharmacology of RCT [2]. However, contrary to what has been achieved in the field of LDL control through statin therapy, pharmacological modulation of HDL biology has not achieved a comparable success in the clinical arena. Nevertheless, this growing burden of knowledge has yielded a new generation of drugs which are under clinical evaluation and are able not only to increase HDL levels and function, but also to achieve a measurable atherosclerotic plaque regression. Within these drugs, apo-AI Milano analogs and CETP (Cholesterol ester transfer protein) inhibitors dalcetrapib and anacetrapib deserve to be highlighted according to the state-of-the-art clinical evidence.

## Reverse cholesterol transport (RCT)

**Figure 1:**
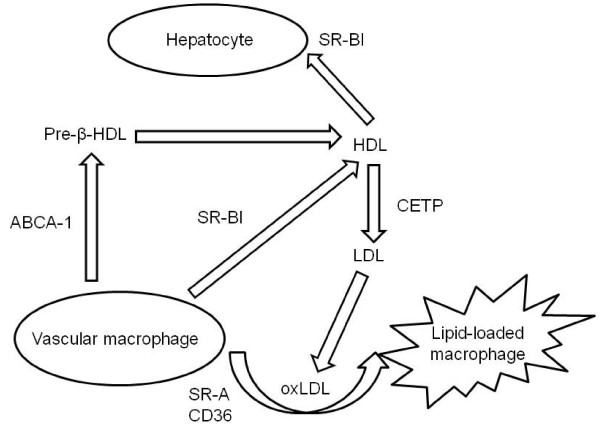
**Simplified scheme of reverse cholesterol transport**. In the onset and progression of atherosclerotic lesions the uptake of modified LDL (mainly oxidized LDL or oxLDL) by macrophages through a process mediated by scavenger receptors (i.e. SR-A and CD36) that lead to the formation of lipid-loaded cells is critical. This seems to be a reversible process, as HDL-mediated RCT can clear cholesterol from vascular tissues contributing to atherosclerosis regression. HDL acquires cholesterol through a mechanism that involves the receptor SR-BI and transports this cholesterol back to the liver. However, HDL also exchanges lipids with LDL, a process mediated by the CETP that increases LDL cholesterol cargo and potentially enhances their atherogenicity.

Early in the 80's it was demonstrated that HDL can act as an acceptor of cellular cholesterol, the first step in the pathway known as RCT [3]. Briefly, HDL precursors (lipid-free apoA-I or lipid-poor pre-β_1_-HDL) are produced by the liver, the intestine or are released from lipolysed VLDL and chylomicrons. PLTP (Phospholipid transfer protein)-mediated phospholipid transfer facilitates apo-AI lipidation and the formation of pre-β-HDL [2]. Lecithin cholesterol acyl-transferase (LCAT) esterifies cholesterol in HDL [4]. Cholesterol esters, more hydrophobic than free cholesterol, move into the core of HDL particle, creating a gradient that enables HDL to accept free cholesterol. After scavenging cholesterol from peripheral tissues, HDL transports cholesterol to the liver where it will be excreted or recycled. The selective uptake of cholesterol esters from HDL into hepatocytes is mediated by the scavenger receptor B type I (SR-BI) [2], and facilitated by the ATP binding cassette (ABC) transporters ABCA1 and ABCG1 [4]. However, cholesterol esters may also be transferred from HDL to other lipoproteins, including chylomicrons, VLDL and LDL, a process mediated by the CETP. Therefore, CETP possesses a potential atherogenic role by enhancing the transfer of cholesterol esters from antiatherogenic lipoproteins (HDL) to proaterogenic ones (mainly LDL). A summary of HDL regulation is shown in the Figure 1.

## Effects of HDL

### Antiatherosclerotic effects of HDL

Atheromatous plaques are not irreversible lesions. Indeed, pioneer experimental studies have demonstrated that HDL administration inhibits development of fatty streaks and induces regression of atherosclerotic lesions in cholesterol-fed rabbits [5,6]. Nowadays the global burden of atheromatous plaques can be measured by novel image techniques. This technology has made it possible to demonstrate that in animal models atherosclerotic plaques are reduced when HDL function is enhanced [7], and that pharmacologic treatments that modulate lipid profile (enhance HDL and decrease LDL) are able to reduce atherosclerosis progression in humans [8].

Given the central role of HDL in RCT, HDL is considered essential in therapeutic strategies aimed to inhibit/regress atherosclerotic lesions [2]. HDL can, therefore, deplete atherosclerotic plaques through their ability to promote efflux of cholesterol from lipid-loaded macrophages [9]. However, HDL is a complex macromolecule containing diverse bioactive lipids and a variety of apolipoproteins and enzymes that could individually contribute to specific antiatherogenic effects [10]. These effects are briefly reviewed in the following sections.

### Anti-inflammatory effects of HDL

Numerous studies suggest that the anti-atherogenic effects of HDL are also related to their anti-inflammatory properties [10,11]. For instance, in macrophages, HDL prevents the conversion of progranulin into proinflammatory granulins [12]; while in endothelial cells, HDL inhibits the expression of cell adhesion molecules VCAM-1, ICAM-1 and E-selectin [13,14]. In animal models, HDL reduces leukocyte homing to arterial endothelium [15], and increased HDL levels have been associated with a decrease of the blood concentration of proinflammatory molecules both in animal models and in patients [16,17].

### Antioxidant effects of HDL

HDL lipoproteins are able to counteract LDL oxidation, which is commonly considered a key event in atherogenesis. HDL inhibits the enzymatic and non-enzimatic oxidation of LDL, and exerts indirect antioxidant effects acting as a "sink" for oxidized products that come from oxidized LDL and transport them to the liver [18]. The antioxidant properties of HDL are attributed not only to apoA-I, the most abundant protein in HDL, but also to several enzymes including paraoxonase (PON), platelet-activating factor acetylhydrolase (PAF-AH) and glutation peroxidase (GPx) [19].

### Antithrombotic effect of HDL

Deregulation of haemostasis plays a key role in acute coronary syndromes. Tissue factor activates the extrinsic coagulation pathway and is essential in atheromatous plaque rupture. HDL is able to reduce thrombin-induced tissue factor expression in endothelial cells [20], to inhibit platelet activation [21], and to stimulate the activation of the anticoagulant proteins C and S [22].

HDL increases the release of two major antiatherogenic/antithrombotic molecules by vascular cells: nitric oxide (NO) and prostacyclin [23]. HDL not only prevents the inhibition of endothelial nitric oxide synthase (eNOS) by oxLDL [24], but also induces *per se *eNOS [25] leading to the increase in NO production by the endothelium.

## Emerging strategies to increase HDL: apo-AI Milano and CETP inhibition

### Genetics of HDL: apo-AI Milano

Apo-AI Milano is a naturally occurring mutant of apo-AI first described in 1980 [26]. Paradoxically, carriers of this mutation have very low HDL cholesterol levels, but no increase in the risk of heart disease. In apo-AI Milano arginine in position 173 has been substituted by cysteine. This extra cysteine confers the capacity to form disulfide bonds yielding homodimers and heterodimers with apo-AI [27]. Several studies in rabbits that received recombinant apo-AI Milano suggest that apo-AI Milano could increase RCT [28], although this has not been observed in mice expressing apo-AI Milano [29].

### CETP and cholesterol efflux to the liver

CETP is synthesized in the liver, spleen, adipose tissue and macrophages, and circulates in plasma bound to HDL. The main function of CETP is to facilitate the transport of cholesterol esters and triglycerides among lipoproteins [2]. It collects cholesterol esters from HDL and exchanges them for triglycerides from chylomicrons, VLDL and LDL, and viceversa (Figure 1). Therefore, the net effect of CETP is proatherogenic. In fact, rare mutations leading to increased function of CETP have been linked to promote CVD [30]. On the other hand, genetic deficiency of CETP leads to marked increase in plasma levels of large HDL particles, and these HDL have enhanced ability to promote cholesterol efflux from foam cells [31]. CETP activity is also inhibited by current lipid-lowering drugs, such as statins, fibrates and niacin [32]. Thus, this molecule may be considered as an interesting therapeutical target in order to prevent cardiovascular disease. However, recent epidemiological studies seem to challenge the mainstream consideration of CETP as a proatherogenic stimulus. In two different large population-based studies, CETP mutation has been associated with greater risk of cardiovascular disease [33,34].

## Emerging drugs to increase HDL function

### Apo-AI mimetics

When combined with phospholipids, recombinant apo-AI Milano (ETC-216) can be given in an intravenous setting. It has been shown to decrease atherosclerotic plaque size in animal models [7]. This effect has also been demonstrated in humans [35]. After five weeks of this intravenous treatment with ETC-216, patients have an increase of HDL, a remarkable decrease of atheromatous burden and a subsequent lower cardiovascular morbimortality [36,37]. In some of these analogs, four phenylalanine residues make a gastro-resistant molecule which enables its oral administration [38]. Notably, considerable debate still exists whether injection of wild-type Apo-AI can be as effective as this natural variant to stimulate RCT [39-41]. A brief summary on the clinical evidence of infusion of apo-AI Milano and wild-type apo-AI is shown in the Table 1.

**Table 1:** Clinical evidence on apo-AI HDL infusion

Study	Patients	Administration	Result
Nicholls et al., [36]	47 post-acute coronary events (randomized)	Intravenous Apo-AI Milano	4.6% decrease of lamina elastic interna (measured by ultrasound)

Nissen et al., [37]	123 post-acute coronary events (randomized)	Intravenous Apo-AI Milano	4.2% decrease of atheromatous plaque volume (measured by ultrasound)

Tardif et al., [40]	183 patients on coronariography (randomized)	Intravenous reconstituted wild-type HDL	3.4% decrease of plaque volume (measured by ultrasound) at follow-up(not difference at baseline)

Shaw et al., [41]	20 patients with claudication who underwent femoral endarterectomy (randomized)	Intravenous reconstituted wild-type HDL	Decreased expression of vascular cell adhesion molecule-1 and decreased lipid content

### CETP inhibition

**Figure 2:**
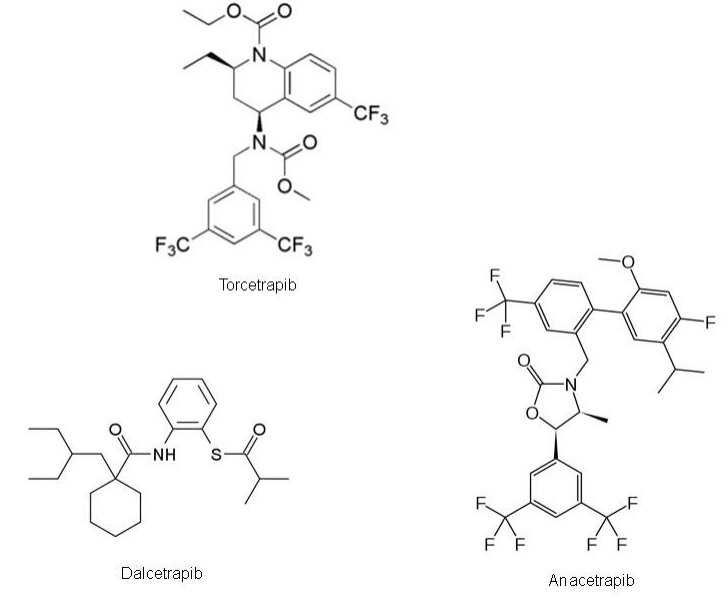
**Chemical structure of torcetrapib, dalcetrapib and anacetrapib**.

Some CETP inhibitors have been demonstrated to possess a significant effect to increase HDL and decrease LDL (Figure 2). Torcetrapib showed an important effect in the rise of HDL in animal models and in the clinical arena. Among phase III studies, the ILLUSTRATE trial (910 patients, double-blind, randomized trial) compared torcetrapib and atorvastatin against atorvastatin alone [42]. It showed that in the highest quartile of HDL increase, a higher atherosclerotic regression measured by ultrasound was observed [42]. However, no benefit was observed in the whole cohort, and a higher rate of high blood pressure was noted in the torcetrapib group [42]. This torcetrapib-linked adverse effect was confirmed in the double-blind, randomized, ILLUMINATE trial (15067 patients, same comparative groups). In this trial, a higher overall cardiovascular mortality was noted in the torcetrapib group despite an efficient HDL increase [43]. This was related to sodium rise, potassium decrease and blood pressure increase, and therefore the study was stopped, the drug being withdrawn in the United States and the European Union [44]. Further studies linked these effects with a torcetrapib-mediated increase in mineralocorticoid stimulation [45]. However, this effect is not mediated by CETP inhibition and therefore is not necessarily shared by other CETP blockers. Therefore, the role of CETP inhibitors dalcetrapib [46] and anacetrapib [47] is being evaluated, given that they seem to increase HDL without the adverse effect in blood pressure. Ongoing big phase III will eventually show the potential of these promising drugs.

Dalcetrapib (Figure 2) offers the advantage of a high pharmacological potency, having 0.4-10 μM IC_50 _for the inhibition of CETP (compared to 19-79 μM of torcetrapib). Dalcetrapib is highly specific for the interaction of cysteine 13 from CETP [32]. Preclinical animal models showed its capability to increase HDL without a significant increase in blood pressure [48]. Further clinical trials demonstrated that it does not possess hypertensive effect in humans and strengthened the idea that dalcetrapib rises HDL without any significant increase in blood pressure [46,49]. Large phase III trials are still ongoing with this drug. The dal-VESSEL trial will assess the effect of dalcetrapib in the reduction of atheromatous plaques measured by PET/TAC and MRI [32]. The dal-OUTCOMES trial will evaluate the effect of the drug on the cardiovascular morbimortality of a sample of 15600 high-risk patients [32].

Anacetrapib (Figure 2) is another CETP inhibitor. It has an IC_50 _of 15-57 μM [32]. Its capability to raise HDL without a parallel increase of blood pressure has been demonstrated in phase I trials [50]. A broader study (589 patients) showed that treatment of anacetrapib and atorvastatin 20 mg increased HDL [51]. Anacetrapib, at the maximum tolerated dose of 300 mg, is able to increase HDL and decrease LDL. However, this last effect is notably enhanced when given with statins, reaching a 70% reduction, without any increase in blood pressure [51]. Based on this synergism strategy, the DEFINE trial will evaluate the role of anacetrapib on 1623 patients whose statin treatment has achieved LDL < 100 mg/dl, but having HDL < 60 mg/dl [32].

### Liver × Receptor (LXR) agonists

There are two LXR isoforms: LXRβ, ubiquitously expressed, and LXRα distributed in a tissue-specific fashion, but mainly in liver and tissues involved in lipid metabolism. LXRs are activated by specific oxidized forms of cholesterol (oxysterols) such as 24(*S*)-hydroxycholesterol and by certain intermediates of the cholesterol biosynthesis. Analysis of LXR-deficient mice has revealed a broad role of this receptor in the regulation of genes involved in lipid homeostasis [52]. For instance, in mice LXR agonists reduce cholesterol absorption in the intestine due to the up-regulation of ABCG5 and ABCG8, which increase the efflux of cholesterol thereby limiting its absoption by intestinal cells [53]. Most interestingly, ABCA1, a key transporter in the efflux of cholesterol and phospholipids from macrophages, is a direct target of LXR [54]. In fact, treatment with endogenous LXR agonists is able to increase the RCT from macrophages and foam cells and thus increase the biliary excretion of this lipid [55]. At the same time, synthetic LXR agonists are able to prevent atheromatous plaque formation in mice [56]. However, these drugs promote the expression of lipogenic genes in the liver, which increase the levels of triglycerides and promote hepatic steatosis [57]. For this reason, research on more selective LXR ligands is an active field of experimental pharmacology.

### Scavenger receptor B-1 (SR-BI) inhibitors

SR-BI is a receptor which modulates the hepatic uptake of cholesterol esters by HDL, VLDL and native LDL. Mice without SR-BI have increased LDL levels and are prone to atherosclerosis [58]. However, in humans SR-BI blockade by ITX5061, a molecule initially characterized as a p38 mitogen-activated protein kinase (MAPK) inhibitor, increases HDL, although this effect seems to be very transient [59].

### Summary

RCT is thought to be one of the most important HDL-mediated cardioprotective mechanisms. However, contrary to what has been achieved with LDL modulation by statin treatment, this mechanism has not been able to be modulated by safe and effective drugs to date. In fact, the beneficial therapeutic effects of raising HDL are proving difficult to confirm in humans. Recent experimental, translational and clinical research has yielded new drugs inspired, in some cases, in variants found in Nature, such as apo-AI Milano analogs, and CETP inhibitors (dalcetrapib and anacetrapib). Low HDL level is the most frequent lipoprotein abnormality in patients suffering premature CVD. In this context, ongoing clinical studies with emerging drugs aimed to increase HDL function have aroused a great interest and opened new expectations in the treatment of CVD.

## Competing interests

Santiago Redondo has given conferences and received honoraria from Gilead and Merck Sharp & Dohme.

## Authors' contributions

CU and TT undertook the initial review of the literature. SR and TT conceived the idea of the manuscript. SR, CU, J M-G and TT took part in the design of the manuscript draft. SR, CU, J M-G and TT built the final version. All authors read and approved the final manuscript.
